# High protein S activity due to C4b‐binding protein deficiency in a 34‐year‐old Surinamese female with ischemic retinopathy

**DOI:** 10.1002/ccr3.1464

**Published:** 2018-03-30

**Authors:** René Mulder, Jeroen K. de Vries, Rogier P.H.M. Müskens, André B. Mulder, Michaël V. Lukens

**Affiliations:** ^1^ Department of Laboratory Medicine University Medical Centre Groningen Groningen The Netherlands; ^2^ Antonius Hospital Sneek The Netherlands; ^3^ Department of Ophthalmology University Medical Center Groningen Groningen The Netherlands

**Keywords:** C4BP, C4BPA, free protein S, p.I300T, p.R240H, retinopathy

## Abstract

In this study, we present the first case of a 34‐year‐old Surinamese female with ischemic retinopathy and increased free protein S due to C4BP deficiency. Possibly, the low PS/C4BP complex level has increased the risk of arterial thrombosis in our patient.

## Introduction

Protein S is a vitamin K‐dependent protein that inhibits coagulation by acting as a cofactor for both tissue factor pathway inhibitor (TFPI) and activated protein C (APC). Therefore, it is not surprising that a deficiency of protein S predisposes to venous thrombosis. In this study, we present for the first time a case of a 34‐year‐old Surinamese female with ischemic retinopathy. After the exclusion of hypertension, diabetes mellitus, vasculitis, hyperviscosity, and sickle cell disease, a thrombophilia workup was performed, thereby discovering an increased free protein S due to decreased complement component C4‐binding protein (C4BP) levels. A thrombophilia workup was performed in the proband. In addition, free protein S antigen and activity were measured in consecutive samples. Free protein S antigen was also measured in father and 1 brother. C4BP levels were measured with an ELISA against the *α*‐chain of C4BP. Next, direct sequencing analysis was performed on C4BPA (encoding C4BP*α*, NM_000715), C4BPB (encoding C4BP*β*, NM_000716), and the sex‐hormone‐binding globulin (SHBG)‐like region of protein S (NM_000313) in the proband. Consequently, possible pathogenic mutations were screened in father and brother to determine segregation. We found increased free protein S and decreased C4BP levels in the proband. Genetic analysis of protein S and C4BP did reveal at least 1 potential pathogenic mutation in C4BPA gene (p.R240H). This mutation was absent in both father and brother who had normal free protein S levels. In this study, we present for the first time a case of a 34‐year‐old female with ischemic retinopathy and increased free protein S due to decreased C4BP levels. Genetic analysis of protein S and C4BP did reveal at least one potential pathogenic mutation in C4BPA gene. Additional screening for these mutations in father and one brother could not exclude the possible association between C4BPA p.R240H and decreased C4BP levels. Furthermore, it remains unclear how the high protein S activity due to decreased C4b‐binding protein is related to the ischemic retinopathy in our patient. The protein S fraction bound to C4BP may have some anticoagulant activity, as suggested before. Maybe, the low PS/C4BP complex level has increased the risk of arterial thrombosis in our patient. Nonetheless, future studies are required on this issue.

Protein S is a vitamin K‐dependent protein that inhibits coagulation by acting as a cofactor for both tissue factor pathway inhibitor (TFPI) accelerating the inhibition of activated factor Xa, and activated protein C (APC) by forming lipid‐bound complexes that accelerate the inactivation of factor Va and factor VIIIa 1, 2. Deficiency of protein S predisposes to venous thrombosis.

In plasma, protein S is present in two forms, a free fraction consisting of 40% of total protein S, which is considered as the active form and the remaining fraction bound to the *β*‐chain of complement component C4‐binding protein (C4BP) 3. Even though it is generally accepted that only the free form of protein S has cofactor activity, the protein S fraction bound to C4BP may have some anticoagulant activity, as suggested before 4. The C4BP protein contains six or seven *α*‐chains and one *β*‐chain linked by disulfide bridges. The *α*‐chains are responsible for binding C4b and the *β*‐chain binds protein S 5, 6. Besides its indirect role in coagulation, C4BP is also involved in regulating the classical and lectin pathways of complement activation by binding activated complement protein C4b 5. During inflammation, the expression of the *α*‐chain increases, without a significant rise in *β*‐chain expression this insures that the level of free protein S is not significantly affected during inflammation 7.

## Case Report

**Figure 1:**
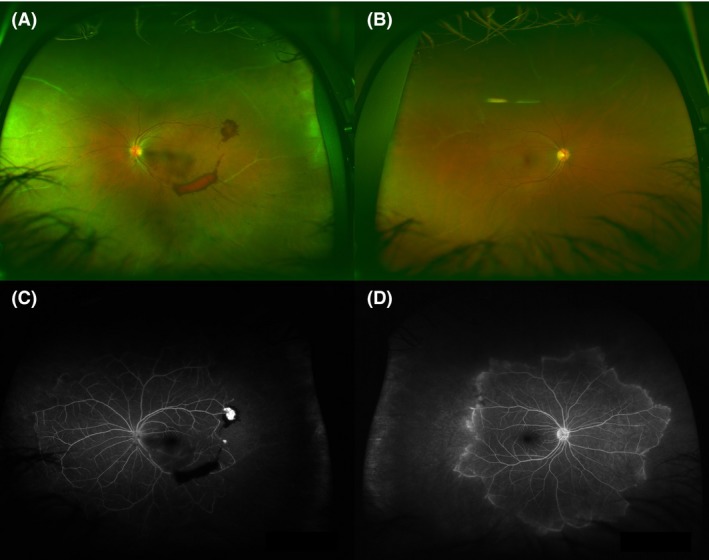
Retinal images. Wide field fundus photography of the left (A) and right eye (B) of the patient showing white occluded vessels in the retinal periphery and a retinal neovascularisation in the temporal superior arcade of the left eye (A), with some pre‐retinal blood. Wide field fluorescence angiography of the left (C) and right eye (D) shows large ischemic areas with complete obliteration of the peripheral retinal vessels in both eyes and hyperfluorescence of the retinal neovascularisation in the left eye (C).

A 34‐year‐old Surinamese female was referred to the Department of Vascular Medicine of our hospital because of right‐sided blurred vision, caused by ischemic retinopathy and neovascularisation (Fig. 1). Her medical history was remarkable for heterozygote alpha‐thalassemia, but negative for thrombotic and recurrent infectious events. The family history was also negative for thrombotic events or bleeding tendency. The patient was not treated with anticoagulants or platelet inhibitors. Physical examination was normal (BMI 23.0 kg/m^2^, blood pressure 125/70 mmHg, heart rate 85/min, normal heart sounds, and no vascular murmurs). After the exclusion of hypertension, diabetes mellitus, vasculitis, hyperviscosity, and sickle cell disease, a thrombophilia workup was performed. All hemostatic laboratory parameters were found normal except for an increased protein S activity (179%) with high free protein S level (207%), despite a normal total protein S antigen level (98%) (Table 1). This suggested that, in contrast to normal conditions, all protein S was free protein S, instead of 30–40% free and 60–70% bound protein S. High activity and antigen levels of free protein S were confirmed in a separate sample 3 months later. Clinically, a protein S deficiency is considered as a possible cause of hypercoagulation, which in turn could have led to the retinopathy. The incidental finding of elevated protein S levels were not considered explanatory of the condition. We did not initiate anticoagulation or thrombocyte aggregation inhibition. The patient's vision did not improve remarkably, nor did it deteriorate in the subsequent months after her initial visit.

**Table 1:** Laboratory results

	Value	Reference values	Units
INR	1.0	<1.3	
aPTT	24	23–33	sec
Fibrinogen	2.3	1.7–4.0	g/L
Antithrombin	91	80–120	%
Lupus anticoagulant	Negative	Negative	
FVleiden/FII G20210A	Negative/negative	Negative	
Protein C activity	100	65–150	%
Protein S total	98	65–150	%
Protein S free	207	60–140	%
Protein S activity	179	65–150	%
C4BP	18	56–168	%
FII	140	60–140	%
FV	98	65–150	%
FVII	92	65–150	%
FX	163	65–150	%
Complement			
Classical pathway	106	69–129	%
MBL pathway	122	70–130	%
Alternative pathway	60	30–113	%

The high free protein S level in the proband was caused by a reduced level of C4BP (18%), as measured with an ELISA against the *α*‐chain of C4BP. To identify a possible molecular defect underlying the C4BP deficiency, we sequenced the protein‐coding regions and exon/intron boundaries of C4BPA (encoding C4BP*α*, NM_000715) and C4BPB (encoding C4BP*β*, NM_000716).

We identified two previously reported non‐synonymous variants in C4BPA, that is, c.719G>A (p.R240H), rs45574833 ((A=0.0140/1686 (ExAC), A = 0.0030/15 (1000 Genomes), A = 0.0095/124 (GO‐ESP), A = 0.0078/227 (TOPMED)), and c.899T>C (p.I300T), rs4844573 (C = 0.4014/47780 [ExAC], C = 0.4898/2453 [1000 Genomes], C = 0.4841/6296 [GO‐ESP], T = 0.4689/13653 [TOPMED]).

**Figure 2:**
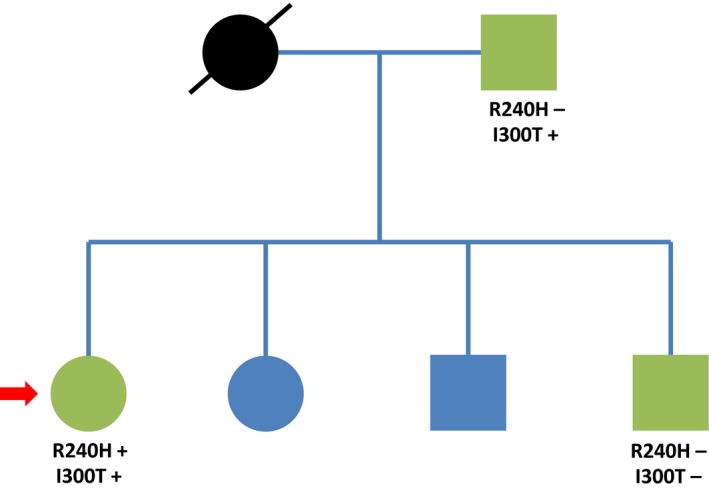
Family tree. Square symbols, male sex; round symbols, female sex; arrow, proband; green symbols, included subjects; blue symbols, not available for inclusion.

In addition, we screened the father and brother for these mutations. Both are also Surinamese. Only p.I300T was present in the father (Fig. 2). Both father and brother had normal free protein S levels. Mother, second sister, and second brother were unavailable for testing.

Discordant data concerning the association between C4BP polymorphisms and atypical hemolytic uremic syndrome have been reported 8, 9. Furthermore, these two variants have also been identified in women experiencing recurrent miscarriages, but due to equally high frequencies between patients and controls, the importance of these variants in this particular clinical situation may be questionable 10. No mutations were found in the C4BPB gene.

Because we could not find clear molecular defect in the C4BP gene responsible for the reduced expression, and a recent report indicated that binding of protein S to C4BP promotes cellular secretion of C4BP 11, we also sequenced the sex hormone‐binding globulin (SHBG)‐like region of protein S (NM_000313), responsible for binding to C4BP using the previously published primers 12, 13. No mutations explaining a reduced binding of protein S to C4BP were found in the SHBG‐domain of protein S.

Although the medical records did not report any use of vitamin K antagonist, we excluded the interference of any form of vitamin K antagonists by the measurement of coagulation factors II, VII, and X which were all normal, except for factor X which was slightly increased (163%) (Table 1). Furthermore, there was no indication of reduced capacity of liver synthesis with normal antithrombin and factor V levels. Furthermore, we also did not find a clear effect of decreased C4BP levels on the complement route, with normal complement pathway activities (Table 1).

## Conclusion

We present for the first time a 34‐year‐old female with ischemic retinopathy and incidental finding of increased free protein S due to decreased C4BP levels. Additional genetic analysis of protein S and C4BP did reveal at least one potential pathogenic mutation in C4BPA gene (p.R240H). Screening for these mutations in father and one brother could not exclude the possible association between this mutation and decreased C4BP levels.

To our knowledge, this is the third report on increased free protein S levels due to decreased C4BP levels 14, 15. However, this is the first study that investigated the molecular background of this phenomenon and revealed two previously published nonsynonymous variants in C4BPA that seem to have no significant effect on the overall expression of C4BPA. In the study of Comp et al.*,* a family is identified with reduced C4BP levels and increased free protein S levels 14. However, no causes for the reduced C4BP levels were reported. The second study reported low C4BP levels with increased protein S activity in neonates 15. These authors propose that the increased protein S activity might be physiological and protect the newborn against thrombosis.

It remains unclear how the high protein S activity due to decreased C4b‐binding protein is related to the ischemic retinopathy in our patient. The protein S fraction bound to C4BP may have some anticoagulant activity, as suggested before 4. Perhaps the low PS/C4BP complex level has increased the risk of arterial thrombosis in our patient. However, further studies are needed on this issue.

## Conflict of Interest

None declared.

## Authorship

RM, JKV, RPHMM, ABM, and MVL: performed the research. RM, JKV, RPHMM, ABM, and MVL: designed the research study. RM, JKV, RPHMM, ABM, and MVL: analyzed the data. RM, JKV, RPHMM, ABM, and MVL: wrote the paper.
